# English linguistic neo-imperialism in the era of globalization: A conceptual viewpoint

**DOI:** 10.3389/fpsyg.2023.1149471

**Published:** 2023-03-08

**Authors:** Jie Zeng, Ariel Robert Ponce, Yuxin Li

**Affiliations:** ^1^School of Foreign Languages, Chengdu Normal University, Chengdu, China; ^2^College of Arts and Letters, Polytechnic University of the Philippines, Manila, Philippines

**Keywords:** linguistic neo-imperialism, linguistic imperialism, language dominance, lingua franca, globalization

## Abstract

As Phillipson warned, “[l]inguistic imperialism [is] alive and kicking” and has become even more subtle in an era when English has become the global *lingua franca*. With this, this conceptual paper aims to propose features of linguistic neo-imperialism by describing how English has continuously spread and retained its power in various domains particularly in periphery countries, whether ex-colonies or non-colonies. Broadly, we highlight these features from the aspects of communication, business, academia, and education. The features of English linguistic neo-imperialism are interrelated and interactive in these fields, reinforcing the current dominant position of English. We then proceed with drawing implications for the local languages, particularly in their preservation and use alongside English and other dominant *lingua franca*s.

## 1. Introduction

Linguistic imperialism is a theoretical construct proposed by Phillipson (1992) that aims to explain the hierarchy of languages, address why some languages are more dominant than others, identify what structures and ideologies facilitate this process, and determine language professionals’ roles. Based on the *Hegemonic Theory* of Gramsci (1971) and Galtung's (1980) *Structural Theory*, Phillipson (1992) pointed out that linguistic imperialism is formed by structural and cultural inequality between English and other languages. Structural inequality refers to inequality related to material wealth, while cultural inequality refers to non-material or ideological inequality. The historical record demonstrates that English imperialism did not emerge anywhere. It is the culmination of a fragmented process that began in the United States after WWII. More than 1.8 billion people who live in core English-speaking countries (e.g., United Kingdom and United States) and the periphery English-speaking countries (e.g., the Philippines, Malaysia, China), according to Phillipson’s framework, speak English throughout the world now (Crystal, 2006). Linguistic imperialism first came into being through colonization and military power. And then more subtly through economic, social, and cultural penetration (Knowles, 1998). Canagarajah (1999) proposed that the current situation of English linguistic imperialism is the product of global English language teaching and discussed the measures to resist English linguistic imperialism.

Phillipson (1992) clarified that the development of linguistic imperialism went through three stages: (1) imposing the colonizer’s power and language; (2) training a group of local elites and privileged classes who serve the benefit of the colonists; and (3) ideological persuasion using media and technology. Linguistic imperialism has already gone through the first two stages in an age of globalization. With the Anglo-American colonial powers’ further retreating from Asia and Africa, it is often questioned whether the concept of linguistic imperialism still applies to explain the prevailing status of English today. As Phillipson (2012) warns, “[l]inguistic imperialism [is] alive and kicking.” The superiority of the English language has been deeply rooted in the ideology of the educated in postcolonial countries or regions. Postcolonial states may have been decolonized politically, but people’s minds may not be (Lai, 2019). This perception, among other factors, poses a significant threat to local languages and contributes to deterioration and eventual language attrition among their speakers (Ahn et al., 2017). Although the notion of linguistic imperialism has paved the way for the discussion of language hierarchization and the factors that reproduce it, there are nuances in how the speakers have consumed and appropriated dominant languages like English that need to be accounted for and may not have been completely explained by the existing theory. Hence, this article aims to propose features of linguistic neo-imperialism in various domains particularly in periphery countries, whether ex-colony or non-colony. This framework is essential in our (re)thinking and understanding of how disproportions between English and other languages are forged and maintained in modern, emerging societies and the present time that is free from colonization and dictatorship. Broadly, we highlight these features from the aspects of communication, business, academia, and education. We argue that English has transformed from a colonial language into a first, second language, or *lingua franca* among speakers from different cultures and may have greatly impacted local languages in bi/multilingual ecologies. Thus, proposing a new notion of linguistic imperialism is imperative to better understand how the transformation of English contributes to its power in these societies. In this article, we first lay out the origin and nature of linguistic imperialism. After, the features of linguistic neo-imperialism will be highlighted and discussed to unveil the dominance of English in various domains. Lastly, we offer implications for the local languages particularly in their preservation and use alongside English and other dominant lingua francas.

## 2. The origin and nature of linguistic imperialism

In the era of colonialism, it was common for colonizers to exclude and eliminate those who did not speak the same language. As far back as the ancient Greeks, people who spoke non-Greek were stigmatized as barbarians, meaning speakers of non-language (Du, 2015). However, the historical relationship between English linguistic imperialism and the colonialism of European and American countries is closer. Colonization is the settlement of a group of people in different parts of the world, often of their own free will but often to the detriment of local people and cultures. Colonization comes from the Latin word *colonia*, meaning farm or settlement. Meanwhile, imperium, derived from Latin *imperium*, refers to the political and military control exercised by the ruling powers over the people and the cultural values and language use in those areas to eventually achieve the goal of cultural colonization of other countries.

Historically, colonizers may adopt different language policies in a colony, such as the differentiation language policy or the assimilation policy (i.e., allowing only privileged classes to speak the colonizers’ language or spreading their language widely in the colony). British and American colonizers generally adopted the assimilation strategy by beautifying English in the colonies, associating the language with civilization, advancement, and progress, among others (Chen, 2011). Colonizers often belittled indigenous languages, leading to a decline in status. It then rationalized this unequal hierarchical order of languages by portraying English as a symbol of rationality and civilization, representing progress, unity, and modernization while belittling other local languages as backward languages that do not possess these characteristics. In this way, it is clear or implied that only by learning English can the colonized absorb the advanced culture, achieve progress and development, and step into modern society. This practice of elevating and beautifying English and belittling other languages is a typical monolingualism that reveals linguistic imperialism’s nature.

As the embodiment of Anglo-American linguistic hegemony, the historical evolution of English linguistic imperialism is closely related to the rise and fall of the hegemony of Britain and America in the existing international political structure (Zeng and Yang, 2022). The British Empire experienced two periods of *mercantile* empire, and *the sun never sets* empire. In contrast, the United States experienced a period of military and political hegemony during the Cold War and a period of economic and cultural hegemony in the post-Cold War. Corresponding to the four periods of British and American hegemony, English linguistic imperialism has also experienced four periods of historical evolution (i.e., military hegemony, geographical hegemony, language policy hegemony, and soft power hegemony; Guo, 2009), and its connotation has been gradually deepened. English linguistic imperialism has a distinct nature of Anglo-American hegemony since its birth and strengthens or weakens with the changing international influence of Britain and the United States. In addition, linguistic imperialism becomes one of the essential forms of cultural hegemony because language is the carrier of culture and closely relates to the dominant position of British and American countries in politics, economy, science, technology, education, mass media, and other aspects, which leads to new inequalities in the world. This pattern parallels the ‘center-periphery’ inequality pattern formed by Anglo-American hegemony.

## 3. The influence of linguistic imperialism theory

The theory of language imperialism has aroused people’s attention to the world language ecology (Xiao, 2009). Like other forms of imperialism, English language imperialism accompanied the hegemony and expansion of The United Kingdom and the United States, threatening the survival and development of indigenous languages of colonies. It diminishes the use and value of minority languages and completely displaces them (Shannon, 1995). Linguistic imperialism’s privileges given to specific languages lead to inequality among languages. Speakers of dominant languages tend to have advantages in education, employment, and social status, while speakers of minority languages tend to be disadvantaged. Regarding linguistic hegemony, the emergence of dominant and inferior languages results in stalemate and compromise between language users. After English gained the dominant position, people in the colonies often pursued the dominant language and gave up or weakened their native languages. Crystal (2002) worries that L1 speakers of the world’s *lingua franca* may have more power than speakers of other languages and fears that weaker languages will disappear. He estimates that 6,000 languages will disappear in the 21^st^ century due to the development and influence of the global *lingua franca* English.

Since Phillipson (1992) put forward the concept of linguistic imperialism, it has attracted extensive attention in sociolinguistic circles, both for and against it. On the whole, the proposal and development of this theory have promoted scholars’ research on language policy, language planning, and the position of English in applied linguistics. The theory of linguistic imperialism is the best example of how language status shapes society and influences language policy (Xiao, 2009). It clearly explains how imperialist languages, past and present – mainly English, French, and Spanish – were promoted in colonial countries through their dominance in economic, political, social, cultural, educational, and other fields. Globalization has made English a paradoxical continuum: it has become the language of imperialism, consumption, market, Hollywood, multiethnic, war and oppression, opportunity, science, social movements, peace processes, human rights, and cross-cultural communication (Guilherme, 2007). The influence of English language imperialism itself has also gone beyond education. No language can replace English in world politics, economy, science and technology, media, scholarly communication, entertainment, etc. English has become an essential determinant of social and economic progress in many countries and a gatekeeper to a higher socioeconomic status for individuals in periphery countries.

## 4. Features of linguistic neo-imperialism in the era of globalization

Linguistic neo-imperialism is a relatively recent concept, clearly a furthering of the earlier framework of Phillipson. Neo-imperialism in itself is a concept developed in the field of economics. Scholars studying neo-imperialism state that it is ‘the final stage of imperialism because significant capital is separated from the product itself and relies on its power to appropriate the benefits’ (Yu, 2020). Following this notion, it can be said that linguistic neo-imperialism departs from the former notion that colonizers impose and determine the use of capital, in this case, their language. Now, imposition and aggression of external forces are no longer at play. The speakers in the postcolonial communities themselves are the ones maintaining the status of once a colonial language.

When commenting on linguistic neo-imperialism, Phillipson (2012, 2013, 2016a) believes that linguistic imperialism is alive and kicking instead of being a thing of the past. The neo-imperialism in the English language is of linguistic hegemony mainly out of political and commercial considerations. Linguistic imperialism is no longer confined to colonial territories in today’s globalized world but maintains and expands its global influence as the world’s *lingua franca*. It is not easy to describe English’s status and current situation in postcolonial and non-colonial countries in globalization’s complex sociolinguistic environment. Compared to the predatory rule of colonizers over the people of occupied areas through land occupation, cultural infiltration, and assimilation during the colonial era, linguistic neo-imperialism in contemporary society is more subtle, particularly regarding overseas education and media influence (Zeng and Yang, 2022). Hence, linguistic neo-imperialism is the hegemonic power of a language motivated by internal dynamism from the ground. In turn, we propose the following as the features of English linguistic neo-imperialism:

1. Locally-driven. The local people themselves have initiated and maintained the status and use of the colonial/imperial language because of the economic value that goes with it.
2. Structurally-motivated. Because of the burgeoning demand of the use a unifying language, social institutions prescribe the use of this language for inclusion and conformity.
3. Colonial/Imperial attitude. Although colonizers have been out of the picture of these ex-colonies for several decades, the people’s psyche still lingers in the superior–inferior asymmetry which is enforced by ‘the role of race as a key differentiating instrument of social control and hierarchization’ (Tupas, 2022). In turn, local people still think that the indigenous languages are inferior (i.e., regional or national in scope) while dominant languages are superior (i.e., international and standard).
4. Normally-actualized. Not only is the use of the (former) imperial language structurally entrenched, but it is likewise made to appear as status quo in the society. It is not made to appear as (neo-)colonial but simply normal.

Graddol (1997) identified specific domains that we clustered into broad fields. For the purpose of this article, we intend to explain the features of linguistic neo-imperialism from the domains of communication, business, academia, and education. The current dominance of English in the world is mainly due to its value as a *lingua franca* for international communication, and the dominant position is mainly reflected in its value as the international language of communication, followed by the commercial value of English itself and its value in academic research, and education. The dominance of English in these fields is not isolated or exclusive but is linked and interrelated. The following subsections exemplify the above features and how they manifest in the specific domains classified by Graddol.

### 4.1. Linguistic neo-imperialism in international communication

English has become one of the major languages for international communication. Estimates by the British Council suggest that 1.75 billion people, which indicates that a quarter of the world’s population speaks English, and two billion people are expected to speak or learn English by 2020 (Ibrahim et al., 2019). In global communications, English has unquestionably become the first-choice language, not only because people worldwide choose it voluntarily. It is also the result of nearly a hundred years of relentless efforts by Britain and the United Ss to promote English worldwide. The British Council’s annual report from 1940 to 1941 pointed out that promoting overseas English education is an important way to establish permanent and good cultural relations with foreigners. The United States was not far behind in promoting the English language. In 1964, the United States established more than 40 English education and cultural institutions worldwide to spread its culture actively, aiming to establish its global leadership (Phillipson, 1992). This expansion and other socioeconomic factors forged the idea that English has become the primary *lingua franca* of choice for individuals who are speakers of languages other than English worldwide (Jenkins, 2019).

With the excellent promotion by Britain and the United States, the place of English as the first language in global communication seems so secure and unshakeable. In international organizations and conferences, English is not only a critical intermediary language for translation but also a *lingua franca*. Many leaders and officials of non-English speaking countries can use English to deliver speeches at international conferences, which shows the extraordinary vitality of English as a language used in international communication. One of the means of linguistic neo-imperialism is that government organizations, non-governmental organizations, and academic institutions play an increasingly important role in promoting English (Phillipson, 2011). One of the prominent international organizations that pronounced the sole use of English among its constituencies is the Association of Southeast Asian Nations (ASEAN). According to Article 34 of the charter, ‘[t]he working language of ASEAN shall be English’ (ASEAN, 2007), which suggests that the medium of communication among its members cannot be the nation’s local languages (e.g., Filipino, Thai, Vietnamese, among others). This deliberate move of the organization is one of the examples where social structures devise a middle ground to make its member communities come together with a unifying language.

Meanwhile, English is also the primary language used by netizens. Users of the most popular social media platforms (e.g., Twitter, Facebook, Instagram) use English as their preferred language of communication. In fact, there are English words that came about because of their use on these social media platforms. Oxford English dictionary listed the following English words coined from social media use. These are netizen, selfie, twerk, tweet, GIF, and others. This phenomenon not only affirmed the use of English in the domain but also expanded its vocabulary, which the users themselves create, and these seem normal and have become part of their linguistic repertoire and communication in cyberspace.

The use of English in most parts of academia, education, business, and even internet discourse determined the standard of international communication in these domains. With these, international communication generally echoed the discourses in these sectors, as can be seen later on, that depict asymmetrical relations between dominant and imperial languages such as English and the local/national languages in non-English speaking regions.

### 4.2. Linguistic neo-imperialism in business

English as a medium of communication in global business is undeniable. However, regarding English as the first language of communication in modern business entirely as linguistic neo-imperialism is a bit of an overstatement. After World War II, the United States gained global influence under various advantages as a rising superpower. With its strong economic power, it maintains and expands its economic influence in the postcolonial countries and other periphery countries, influencing the system construction and operation of the worldwide economy and trade. Considering the economic attribute of language, English, as the first language in international trade, has been endowed with great economic value (Grin, 2001), and this value is constantly strengthened by the efforts of Britain and the United States to promote the language. Global trade volume growth has also objectively increased the commercial value of English. While enjoying the convenience brought by English in business communication, the peripheral English-speaking countries are also affected by the new and subtle form of linguistic imperialism and even unconsciously cooperate with this linguistic neo-imperialism. In fact, there is an initiative to strengthen and innovate the teaching of business English in countries where English is not the primary language of trade and business (e.g., China) which is aimed at a ‘globalized business context’ (Ai et al., 2018).

Phillipson (2009) posits that linguistic imperialism has progressed into a new stage in the current era. The linguistic neo-imperialism, which is now partly driven by commercial objectives, maintains English hegemony and tries to protect the massive economic interests of Britain and the United States through linguistic inequality and the advantage of English. Since the 1930s, the British Council, for example, has worked to promote the use of English worldwide. It now serves more than 100 countries with consulting services and participates in English language instruction and testing, accounting for three-quarters of its revenue (Du, 2015). According to the British Council’s annual report for 2019 to 2020, the organization’s overall revenue has increased to £1,289 million (British Council, 2021). This promotion has also deconstructed the minds of those in the periphery English-speaking countries. Wang and Hatoss (2022) found that the local language in a Chinese community has been marginalized in the context of trade and ideated English and Putonghua as the ‘capital in the domestic and global markets.’

Meanwhile, the use of English is also at the fore in multilingual workspaces or businesses whose nature is communicating to various target markets (e.g., Business Processing Outsourcing). According to Angouri and Miglbauer (2014), ‘English has become the uncontested language of business in the context of a globalized workplace.’ Because of the presence of multilingual and multicultural human resources in international companies and the prospective market, it is normal that English becomes the dominant language and *lingua franca* in communication; hence, in these contexts, English is expected as the working language.

### 4.3. Linguistic neo-imperialism In academia

In academia, English has become the first language of publication, with most papers published in this language. English’s status as a global academic *lingua franca* is not just because of linguistic neo-imperialism but the result of a combination of factors, including excellent research resources, the development of bibliographic databases and citation indexes, and the long history of the English academic language. Major scientific and academic journals are published in English. A ranking of the use of English in authoritative international journals in the last decade by Rao et al. (2020) shows that the proportions of those English periodicals included in the SCI, SSCI, and A&H Citation Index are, respectively, 98.05, 96.17, and 75.26%. On the other hand, Scopus, an abstracting and citation database, detailed the minimum criteria for indexing journal titles. One of which is that ‘[t]he title should have English language abstracts and article titles’ (Elsevier, n.d.). In effect, those journal titles and articles written in a language other than English cannot penetrate one of the most coveted journal indexing. This is the case because indexing like this is powered by the leading publisher, Elsevier, and complemented and sometimes rivaled by other big publishers like Taylor & Francis, Springer Nature, Black & Wiley, and SAGE. According to Hagve (2020), these publishers have a market share of more than 50% where Elsevier has the biggest, with about 16% market share. And that these academic publishers have global sales of more than $19 billion, positioning them between the music and film industries. As an industry, these publishing houses are unique in their profitability, generating huge net profits and increasing yearly profits. The profits of the academic publishing industry have driven publishers to promote English-language publications relentlessly, reinforcing the dominance of English and deepening the linguistic neo-imperialism in academia.

Because these publishers mainly accept papers written in English and the competition among universities for international recognition and ranking, teachers and researchers use English to publish papers in international journals, especially the high-level papers collected by well-known retrieval systems. This publishing further cemented the dominance of English in academic publishing. To examine the extent to which English has become the dominant language of scientific communication and what motivates users of English to publish their research in English rather than their mother tongues. Stockemer and Wigginton (2019) found in a survey of more than 800 authors of scientific papers published in Springer Nature that non-English-speaking researchers, on average, wrote 60% of their papers in English. The proportion varied by discipline, region, and age group, with younger academics, Europeans, and academics in the natural sciences more likely to use English. They believe that publishing in English will enhance the reputation of one’s work, which is the primary motivation, rather than institutional pressure on researchers to publish in English. In addition, Hamel (2007, p. 54) observed that ‘[…] vernacular languages rarely appear in the debates about languages in science, since their status and corpus are considered unfit to express scientific thought and research findings’. This bias deliberately positions local languages as inferior and incomplete in the context of scientific expression.

However, the use of English is not only driven by structural bodies in academia. Writing in English is normalized because most researchers are actually trained in writing in this language due to the use of English in their research classes at the university level and as the language of science. In turn, research writing and publication have naturally used English as the language in the writing domain.

### 4.4. Linguistic neo-imperialism in education

After the colonial period, many people in postcolonial countries regard English as a stepping stone to success, a gatekeeper to higher education and social status (Sibayan and Gonzalez, 1996; Lai, 2019). In terms of education policies and directions in peripheral countries, English has taken a dominant position in some aspects, which are mainly reflected in the following aspects. First, English is set as a mandatory condition for selecting any higher talent, and the level of English proficiency becomes an important screening criterion. Most universities whose medium of instruction is English require considerable scores on standardized tests. For example, the majority of these universities prescribe TOEFL or IELTS certification to prove foreign and sometimes domestic students’ and teachers’ English language competence for university admission.

Second, many schools advocate teaching in English regardless of subject content. For instance, English education in the Philippines, Malaysia, and Singapore is accompanied by English course contents from primary school to the post-graduate level. Despite the multilingual communities or non-English language ecologies of these Asian countries, their educational systems have patronized the use of English as the medium of instruction, and English is one of the contents taught across levels.

Despite the retreat of Anglo-American colonial powers, the British- and American-inspired higher education systems survived in most postcolonial countries, and English remained the dominant language of instruction in these systems. Even in non-English speaking peripheral countries, many have put forward the goal of building world-class universities, in which an increasing number of courses are taught in English by teachers with overseas education backgrounds, and this trend is increasing.

These accommodations of the status of English in education have received various support and positive attitudes towards its use, especially from the stakeholders despite the retreat of the colonial rule and the advocating of multilingual education (Lai, 2001; Choy and Troudi, 2006; Bokhorst-Heng and Caleon, 2009; Mahboob and Cruz, 2013). The educational systems have reinforced the power of English, which has resulted in the perceived practical use of English as the language used to talk to foreigners (Ponce and Lucas, 2021) and as an index of intelligence (Bacon and Kim, 2018); hence, the people, in general, maintain the status of English as the language of education for the most part because of these notions.

English language education plays a crucial role in social and economic development, whether from the perspective of individual development needs or enterprise and national development strategy. Learning a language, especially English, is a human capital investment with real value. It helps people acquire more knowledge and information, acquire more human capital, and bring more economic benefits. In the neocolonial era, language education gradually replaced the old exploitation methods and served the interests of core countries by creating mainstream discourse systems to establish and consolidate structural and cultural inequalities between English and other languages. Educators should guard against linguistic neo-imperialism in periphery countries when promoting English language education.

So far, we have illustrated how English has established its presence and stayed relevant in the major domains as a result of linguistic neo-imperialism (see Table 1 for the summary). Moreover, the features of linguistic neo-imperialism we proposed here can be treated as an early-stage framework for how we (re)think and understand a dominant, powerful language like English that brought with it both opportunities for its speakers and institutions and threats to other cultures and languages. Perhaps, we consider this framework an emerging one because of the political and economic circumstances that will further shape the understanding of these inequalities as time goes on. Thus, future developments and editions of this framework will be possible.

**Table 1:** Summary of the Features of English linguistic neo-imperialism and its exemplifications in the major domains.

Features of linguistic neo-imperialism	International communication	Business	Academia	Education
1. Locally-driven The local people themselves have initiated and maintained the status and use of the imperial language because of the economic value that goes with it	English has become the primary *lingua franca* of choice for individuals who are speakers of languages other than English worldwide (Jenkins, 2019).	Strengthening of Business English in education for ‘globalized business context’ (Ai et al., 2018)	Researchers and faculty members strive to write papers in English to conform to journal instructions on using English in their manuscripts.	Many people in postcolonial countries regard English as a stepping stone to success, a gatekeeper to higher education, and higher social status (Sibayan and Gonzalez, 1996; Lai, 2019)
2. Structurally motivated Because of the burgeoning demand or the use of a unifying language, social institutions prescribe the use of this language for inclusion and conformity	One of the prominent international organizations that pronounced the sole use of English among its constituencies is the Association of Southeast Asian Nations (ASEAN). According to Article 34 of the charter, ‘[t]he working language of ASEAN shall be English’ (ASEAN, 2007).	English, as the first language in international trade, has been endowed with great economic value (Grin, 2001)	Publishers and indexing markets require journals to have English titles and abstracts (Elsevier, n.d.).	Despite the multilingual communities or non-English language ecologies of these Asian countries, their educational systems have patronized the use of English as the medium of instruction, and English is one of the contents taught across levels.
3. Colonial/Imperial attitude Colonial/Imperial attitude. Although colonizers have been out of the picture of these ex-colonies for several decades, the people’s psyche still lingers in the superior–inferior asymmetry which is enforced by ‘the role of race as a key differentiating instrument of social control and hierarchization’ (Tupas, 2022). In turn, local people still think that the indigenous languages are inferior (i.e., regional or national in scope) while dominant languages are superior (i.e. international and standard).	The use of English In most parts of academia, education, and business sectors determined the standard of international communication in international laws, international translations, and trade. With these, international communication generally echoed the discourses in these sectors that depict asymmetrical relations between dominant and imperial languages such as English and the local/national languages in non-English speaking regions.	Local languages are deemed to have no value in the global market compared to English (Wang and Hatoss, 2022)	Hamel (2007, p. 54) observed that ‘[…] vernacular languages almost never appear in the debates about languages in science since their status and corpus are considered unfit to express scientific thought and research findings’. This deliberately positions local languages as inferior in the context of scientific expression.	The educational systems have reinforced the power of English, which has resulted in the perceived practical use of English as the language used to talk to foreigners (Ponce and Lucas, 2021) and as an index of intelligence (Bacon and Kim, 2018)
4. Normally-actualized Not only is the use of the former imperial language structurally entrenched, but it is likewise made to appear as status quo in society. It is not made to appear as the (neo-)colonial but simply normal.	The use and expansion of English words are made faster because of their internet use. This phenomenon not only affirmed the use of English in the domain but also expanded its vocabulary which the users themselves create, and these seem normal and have become part of their linguistic repertoire.	‘English has become the uncontested language of business in the context of globalized workplace’ (Angouri and Miglbauer, 2014)	Most of the researchers are actually trained in writing in English as a result of the use of English in their research classes at the university and as the language of science. In turn, research writing and publication have naturally used English as the language in the writing domain.	These accommodations of the status of English in education have received various support and positive attitudes towards its use, especially from the stakeholders (Lai, 2001; Choy and Troudi, 2006; Bokhorst-Heng and Caleon, 2009; Mahboob and Cruz, 2013).

For the time being, we want to offer a discussion on whether linguistic neo-imperialism, particularly in the English language, could and should be combated. In the next section, we will offer our insights and propose how to deal with the challenges that it poses.

## 5. Can and should we combat English linguistic neo-imperialism?

As Phillipson (1992, p. 53) points out, opposition to English’s growing dominance came from various sources, including ‘the colonized people, European parliaments, and intellectuals from both the Center and Periphery countries.’ These demonstrators see indications of language imperialism and dominance and want to fight it. However, at this time, English is localized in many countries in the outer circle and is developing and gaining prominence in the countries in the expanding circle. Thus, is it still prudent to combat the spread and use of English, or can we really combat its spread? With the features of linguistic neo-imperialism presented above and its realization in every primary domain, it may be nearly impossible to stop or even slow down the spread and use of English in different speech communities and domains.

The linguistic neo-imperialism of English brings adverse effects on peripheral countries. Firstly, in the face of the spread of English and the penetration of influential western culture, peripheral countries’ traditional cultures and languages are seriously challenged, and national and cultural security is threatened through language shift and, eventually, language loss. One of the ways to protect languages is to mitigate the negative impact of globalization by giving the communities a stronghold on language program initiatives (Stroud, 2003). More broadly, it is essential for a bottom-up approach in carrying out such an initiative. Moreover, people in the locality should be the ones planning how language and culture preservation should be done. Fettes et al. (1998) argued that if members of speech communities are involved in planning and implementing their language programs, these will likely flourish, compared with the top-down approach in language program implementation. Hence, there will be ownership and meaning for these members when they engage themselves in documenting their local languages and dialects as one of the many practical ways to safeguard them.

Moreover, peripheral countries adopted countermeasures such as bilingual or multilingual policies and maintained and propagated their languages and cultures due to the adverse effects on local languages. The European Union emphasized laws and policies (e.g., *European cultural convention, 1977; Treaty of the European Communities, 1992; Treaty of Amsterdam, 1997; Bologna Declaration, 1999*) to maintain the importance of multilingualism and formulate relevant policies to weaken the hegemony of English linguistic neo-imperialism. In the Philippines, the education bureau institutionalized *Mother Tongue-Based Multilingual Education (MTB-MLE)* in 2009 and mandated teaching 19 Philippine languages from kindergarten to third grade*.* Meanwhile, China has also adopted laws and policies such as *The Common Spoken and Written Language Law (2001)* and *The Outline of the National Medium–- and Long-term Program for The Reform and Development of Spoken and Written Languages (2012–2020)* to implement the policy of building a harmonious language life, language protection, language services and improving the national language ability.

On the other hand, initiatives to foster linguistically inclusive cyberspace and social media applications in the globalized internet age should develop features that promote diversity and inclusivity of their users. For example, Facebook provides 70 available translations for non-English speakers (Singh et al., 2012), and other platforms can follow suit. This enables more nuanced online sharing and symbolically acknowledges the presence of other languages and non-English speakers in a platform where there is a very high appraisal for English.

Admittedly, the initiatives abovementioned for local languages to take their place in social institutions and try to keep up with the popularity of English are not new. These have been advocated and implemented in most communities. Nevertheless, at this point, it is also essential to look at these programs and examine their effectiveness (e.g., Li and Majhanovich, 2010; Metila et al., 2016; Feng and Adamson, 2019) in actually carrying out a desired outcome, that is, to make local languages relevant and practical. More importantly, people should start accepting that English is part of the linguistic repertoire of the locals and may index local identity. Therefore, English, like any other local language, should be developed locally and progressively so that people recognize their variety as their own and must be documented like any other language.

## 6. Conclusion

Linguistic imperialism at first sounds like a conspiracy theory, and Philipson (2016b) compares English to a hydra in that it expands at the expense of and destroys other languages, which sounds alarming. Some scholars have criticized the theory of linguistic imperialism (e.g., Fishman et al., 1996; Spolsky, 2004; Pervaiz et al., 2019) as a conspiracy theory that emphasizes the center countries’ imposition and exploitation of the periphery countries. However, in the reality of the present time, English’s supremacy results from a bottom-up movement led by its speakers, and this paper has put forward the features of linguistic neo-imperialism that discuss how English maintained a status, especially in major domains where the idea of force and compulsion from the colonizers are no longer the driving force why English thrives. The proposed framework in this article is a breakthrough in understanding the current dynamics that influence the staying power of English in modern (postcolonial) societies. Hence, future developments of the features discussed above are susceptible to edition and expansion depending on the socio-political events that may arise.

Consistently, the global popularity of English poses a challenge to establishing the international discourse power of other languages. However, the notion of World Englishes, which recognizes various varieties of English around the globe, challenges the Anglo-Saxon ownership of English. In this context, it is timely for speakers of languages other than English to rethink the nature of English globalization, re-examine the status and function of English as a *lingua franca*, and reposition the relationship between English and other languages in language policy and language planning. English is no longer the patent of the core countries but the commonwealth in world communication and even an index of local, multilingual identity. It provides one convenient method of communication for people in the world.

The governments should double time and recalibrate their measures based on the assessments of the current undertakings to ensure that local languages are protected and coexist with English. For one, the government should build greater confidence among its citizens regarding languages and culture; that is, even local languages can yield economic gains and build international relationships besides just being indices of local identities. On the other hand, the government should reformulate and strengthen multilingual education policy and introduce different varieties of English in the educational system. This way, the relevance of local languages and ownership of local varieties of English are ensured. As Crystal (2003) points out, political strength can decide a language to become an international language, but only economic strength can maintain the status of the language and expand its influence.

Finally, we note that, in this article, we capitalized on relevant literature in coming up with exemplifications vis-à-vis the features of linguistic neo-imperialism. The task now is for other scholars to build on this framework to legitimize or even dispute what has been said of linguistic neo-imperialism through case studies and empirical data.

## Author contributions

JZ contributed to conception, design of the study, and wrote the first draft of the manuscript. AP input on the draft and organized additional literature. AP and YL wrote sections of the manuscript. All authors contributed to the article and approved the submitted version.

## Funding

This paper was supported by the Humanities and Social Sciences Youth Foundation of the Ministry of Education of China (Project name: A dialectical study of English linguistic imperialism in the Philippines from the perspective of Belt and Road Initiative, Granted Number: 18YJC740006).

## Conflict of interest

The authors declare that the research was conducted in the absence of any commercial or financial relationships that could be construed as a potential conflict of interest.

## Publisher’s note

All claims expressed in this article are solely those of the authors and do not necessarily represent those of their affiliated organizations, or those of the publisher, the editors and the reviewers. Any product that may be evaluated in this article, or claim that may be made by its manufacturer, is not guaranteed or endorsed by the publisher.
